# Effectiveness of shoulder symptom modification approaches in managing patients with frozen shoulder: study protocol for a randomized sham-controlled trial

**DOI:** 10.1186/s13063-026-09661-z

**Published:** 2026-04-16

**Authors:** Ayesha Seemab, Watson Arulsingh, Ramprasad Muthukrishnan, Praveen Kumar Kandakurti, Roohin Saiyad

**Affiliations:** 1 Physiotherapy Division, College of Health Sciences, Gulf MedicalUniversity, Ajman, United Arab Emirates; 2 Physiotherapy Division, College of Health Sciences, Gulf MedicalUniversity, Ajman, United Arab Emirates; 3 Thumbay Physical Therapy and Rehabilitation Hospital, Ajman, United Arab Emirates

**Keywords:** Frozen shoulder, Adhesive capsulitis, Symptom modification, Manual therapy, Mobilization with movement, Sham mobilization, NPRS, SPADI, Range of motion

## Abstract

**Background:**

Symptom modification approaches in musculoskeletal physiotherapy practice reduce pain and enhance movement. In our study two symptom modification approaches, shoulder symptom modification procedure (SSMP) and mobilization with movement (MWM) along with sham mobilization are compared. This study aimed to compare the effectiveness of the above approaches in managing pain and shoulder disability and improving shoulder movement in patients with frozen shoulder.

**Methods:**

A total of 36 patients with frozen shoulder, will be randomly allocated for 8 weeks into three groups: (a) the SSMP group (n = 12) (b) the mobilization with movement (MWM) group(n = 12) and (c) sham mobilization group (n = 12). Patients with frozen shoulder aged 40–65 and meeting the inclusion criteria will be eligible. Each group will receive 3 sessions in a week for 8 weeks along with different exercises including isometric, eccentric and concentric followed by the functional program. Measurements will occur at four time points: before the initiation of treatment sessions (week 0), followed by 12 treatment sessions (week 4), then 18 treatment sessions (week 6) and two months from the beginning of the trial (week 8). The primary outcome was functional disability (SPADI). Secondary outcomes included pain intensity (NPRS), active shoulder range of motion (ROM), and the Patient Global Impression of Change (PGIC).

**Discussion:**

This pilot study is the first to examine the effectiveness of symptom-modification approaches in patients with frozen shoulder. The study aims to provide preliminary data on feasibility, safety, and early clinical outcomes, as well as to document challenges encountered during implementation. Findings from this investigation will inform the design, methodology, and sample size estimation for a future large-scale randomized controlled trial assessing the efficacy of symptom-modification interventions in this population.

**Trial registration:**

Clinical Trials.gov NCT06763601. Registered on 12/11/2024. URL of trial registry record: https://register.clinicaltrials.gov/prs/beta/studies/S000F7NU00000067/recordSummary.

**Supplementary Information:**

The online version contains supplementary material available at 10.1186/s13063-026-09661-z.

## Background

Frozen shoulder is a common disorder of the shoulder, which is characterized by shoulder pain, stiffness, and limited active and passive range of motion. This is an inflammatory condition with pain and limitation of mobility that could lead to atrophy of the shoulder muscles resulting from disuse [1], commonly known as frozen shoulder, adhesive capsulitis, and later termed periarthritis by Codman [2].

Globally the prevalence of population affected with frozen shoulder is estimated between 2–5%. Frozen shoulder is a common shoulder condition distinguished by shoulder pain, stiffness, and limited active and passive range of motion, manifesting as a progressive loss of glenohumeral movements associated with pain symptoms [3] must have been stabilized for at least one month or deteriorating, persisting for several months up to two years [4]. Pain and stiffness are the manifestations of progressive fibrosis and contracture of the glenohumeral joint capsule [5]. Frozen shoulder normally develops between the age of 40 and 60 years and is more common among females [6]. In the field of physiotherapy, various treatment approaches have been proposed for the management of adhesive capsulitis, including proprioceptive neuromuscular facilitation (PNF), the Spencer technique, Mobilization with Movement (MWM), as well as conservative interventions such as biophysical agents and therapeutic exercises [7–9]. The Shoulder Symptom Modification Procedure (SSMP), introduced by Jeremy Lewis in 2009, is a structured assessment and treatment approach that emphasizes understanding how specific movements influence shoulder symptoms, rather than focusing exclusively on a pathoanatomical diagnosis [10].

As a novel approach in manual therapy, SSMP provides a staged approach of controlled and segmental mobilisations of shoulder complex i.e., thoracic, scapula and humeral head procedures incorporated with functional movements. This approach aims to modify shoulder arthrokinematics mechanisms to achieve pain free movement of the shoulder and optimize shoulder function, making it a valuable clinical tool in the management of shoulder disorders.

Similarly, MWM is a symptom modification technique designed to reduce pain and restore joint mobility. It has gained widespread clinical utilization due to its demonstrated efficacy in the treatment of adhesive capsulitis. Notably, a systematic review conducted in 2022 concluded that manual therapy interventions, including MWM, are associated with clinically meaningful benefits for patients presenting with shoulder pain and functional limitations [11].

In contrast, sham mobilization is employed in clinical trials as a placebo control to isolate and assess the true efficacy of active manual therapy techniques [12]. The use of such a control is critical for understanding the specific effects of symptom modification approaches and their role in the rehabilitation of musculoskeletal disorders.

Although MWM has shown promising results in clinical trials compared to sham mobilization, the effectiveness of SSMP in reducing pain and disability and in enhancing quality of life for patients with adhesive capsulitis remains an area requiring further investigation. The present trial aims to evaluate the potential benefits of SSMP as a targeted, non-invasive intervention for the management of adhesive capsulitis and to compare its outcomes with those of MWM and sham mobilization.

## Objective

The primary objective of this study was to evaluate the effectiveness of SSMP compared with Mobilization with Movement (MWM) and sham mobilization in patients with frozen shoulder for improving functional disability, reducing pain, and improving range of motion.

## Methods

### Study design

**Fig. 1:**
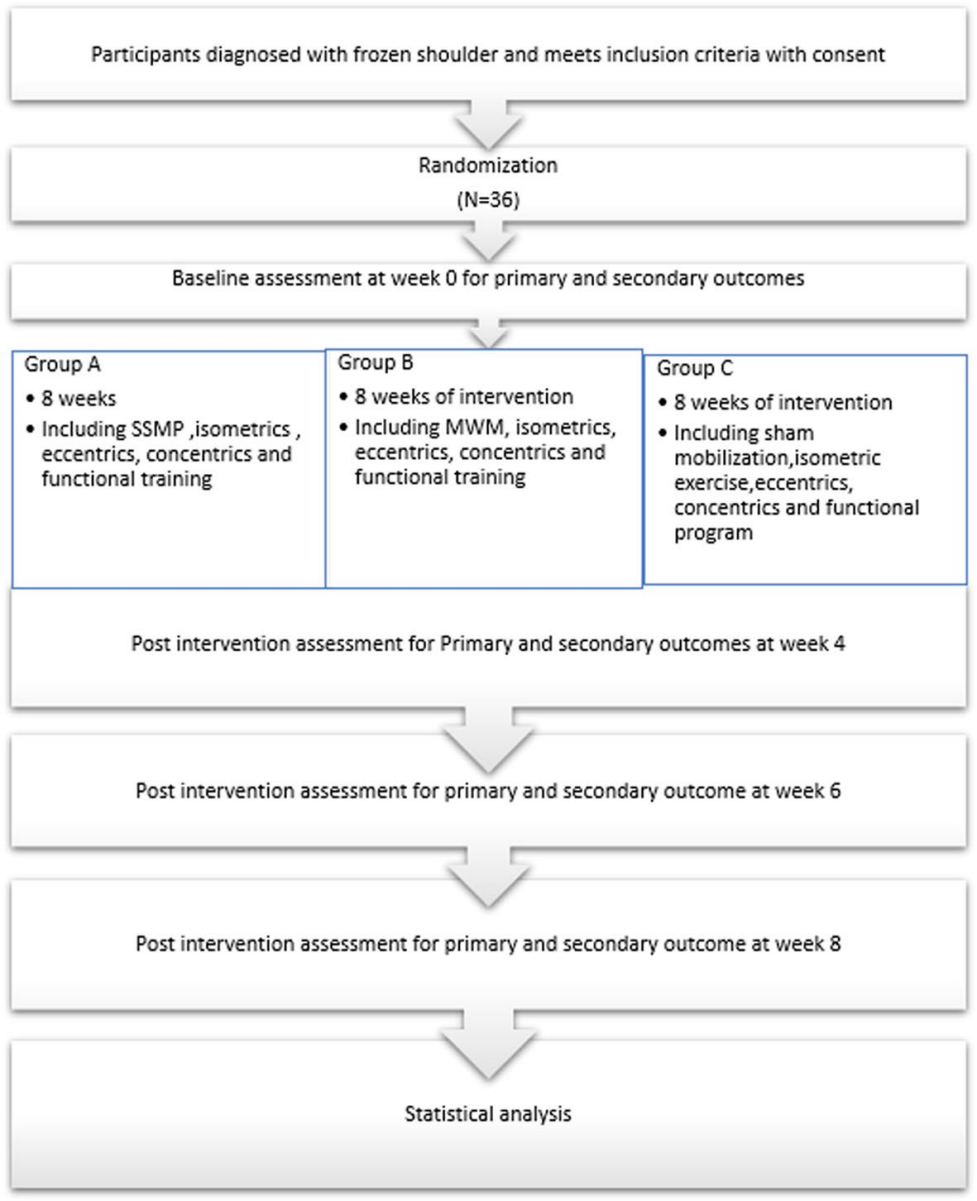
Flow chart of the trial in accordance with the SPIRIT statement

The present RCT is a pilot feasibility study, conducted in accordance with the CONSORT statement. This RCT followed the SPIRIT. (Standard Protocol Items: Recommendations for Interventional Trials) statement [13]. The completed SPIRIT checklist table and figure of the study are added in Annexure. The study flowchart is shown in Fig. 1.

### Setting

The study will be taking patients reporting shoulder pain and diagnosed with adhesive capsulitis in Thumbay Physical Therapy and Rehabilitation Hospital. Ajman and Thumbay Hospital, Ajman in the physiotherapy department, UAE.

### Recruitment

The participants will be recruited from Thumbay Physical Therapy and Rehabilitation Hospital and physiotherapy department of Thumbay Hospital, Ajman and physiotherapy department of Thumbay Hospital Ajman. All participants that will be patients diagnosed with adhesive capsulitis by the orthopaedic physician and referred to the physiotherapy department of the hospital will be considered. The Primary investigator and the two therapists in the same hospital will implement the techniques SSMP, MWM and sham mobilization on patients. All participants will read and sign the consent form. The consent declaration will address the protection of personal data and the rights of participants. Volunteers will be informed about: (a) the purpose, procedures, and specifics of the study, (b) their participation being entirely voluntary, and (c) their ongoing right to withdraw from the study at any time, even after providing written consent.

### Randomization and blinding

Sequence generation will be conducted by a biostatistician and allocation concealment managed by a site coordinator, both of whom are independent of recruitment and treatment delivery. Treating physiotherapists are blinded to group allocation during baseline assessment, and outcome assessment will be performed by a blinded assessor.

The hospital provides dedicated administrative and logistical support through a separate wing, allowing the PI to supervise compliance and protocol adherence without influencing randomization or treatment allocation, ensuring methodological integrity. The primary investigator (PI) will oversee the allocation process but will not participate in patient screening, allocation or treatment delivery, in order to minimize potential selection and performance bias.

### Eligibility

The study population will be the patients who are diagnosed with frozen shoulder. The inclusion and exclusion criteria for participants is given below.

#### Inclusion criteria

- Diagnosis of adhesive capsulitis confirmed by a healthcare professional.
- Scores between 2 and 6 in adhesive capsulitis (AC) scale [14].
- Range of motion affected in capsular pattern.
- Age between 40–65 years.
- Willingness to participate by signing informed consent.

#### Exclusion criteria

- Having any history of shoulder dislocation
- Humerus fractures or trauma
- Having history of cervical radiculopathy
- Diagnosed with any sort of cancer
- History of cardiovascular disease
- Use of glucocorticoid injections

### Intervention

Eligible participants will be randomly assigned to one of three groups: the SSMP (group A), MWM (group B), and Sham Intervention (group C). Each participant will undergo baseline assessment, and after assessment, they will be randomised and allocated into one of the three groups and will receive a four-phase designed specific rehabilitation procedures over an 8-week, followed by a follow-up session to assess improvements.

#### Phase 1—Symptom modification approaches and sham mobilization

After the assessment and allocation in Group A, patient identified concerned (PIC) movement will be observed that includes the most painful movement and then targeted SSMP techniques (thoracic repositioning, scapular facilitation, humeral head mobilization, and neuromodulation) will be introduced based on individual’s PIC movement [10, 15].

Participants in Group B will commence mobilization with movement strategies focusing on restoring active and passive range of motion. MWM technique will be carried out based on PIC movement either in flexion, abduction, external and internal rotation directions. MWM technique will be implemented in 3 sequences of 10 repetitions with a rest interval of 30 s between each sequence [16]. Group C will receive a sham mobilization with movement consisting of hand placements mimicking therapeutic positioning, without applying therapeutic force and will be asked to perform the PIC movement up to the pain free range and the dosage will be the same as that of group B. Placebo is not just a comparison intervention, but also acts as an active mechanism that could partly explain the treatment effects seen with manual therapy [17].

#### Phase II – Isometrics

In this phase all the participants in each group will perform isometric exercises using a metronome set at 1 beat per second with a 3 s hold followed by a 3 s rest, of 4 repetitions.

#### Phase III—Eccentrics and Heavy Slow Resistance exercises

This phase includes eccentric exercises initially performed on the unaffected and then affected by choosing the most painful movement, using a 5 s contraction guided by a metronome. Participants will be made to perform three sets of five repetitions with gradually progression to heavy slow resistance exercise.

Heavy slow resistance (HSR)- the HSR program is a combination of eccentric and concentric loading. One repetition maximum was determined for the concentric loading. Exercise will be performed using a metronome of 3 beats for the eccentric phase as well as 3 beats for the concentric phase, making the eccentric-concentric contraction total 6 seconds per repetition, beginning at 70% of 1 repetition maximum (1RM) and progressing up to 85% of 1RM, with one set of five repetitions.

#### Phase IV—Functional programme

**Fig. 2:**
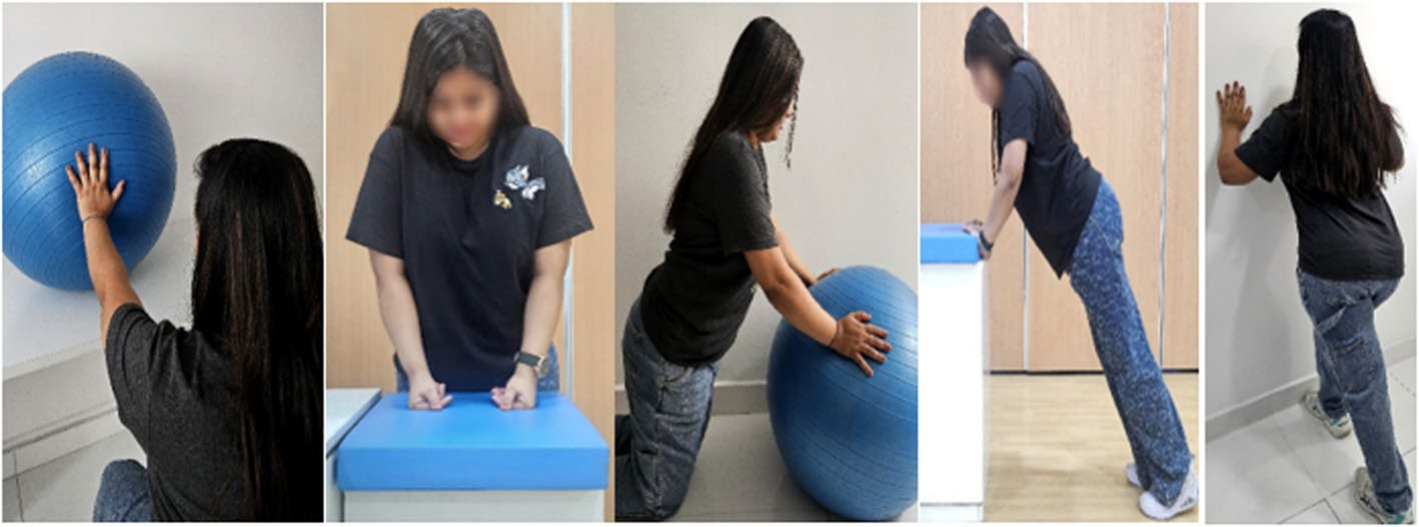
Exercises of functional program

This phase will include five functional exercises, such as wall push-ups and upper limb weight-shifting exercises (as illustrated in Fig. 2). The aim is to restore functional movement patterns relevant to daily activities.

Transcutaneous Electrical Nerve Stimulation (TENS) will be permitted as a standardized rescue analgesic intervention during the first two weeks only if a participant reported an NPRS score equal to or higher than the previous session. The TENS parameters (frequency, pulse duration, intensity, and treatment duration) will be standardized across all groups to ensure uniform exposure. The frequency of TENS usage shall be documented and later incorporated into statistical analysis as a covariate to minimize any confounding effects. TENS The frequency of TENS application will be recorded across all groups.

### Outcome measures

The evaluations will take place at four time points: prior to treatment (week 0), post-treatment (week 4 and 6), and two months subsequent at week 8. The primary outcome of this study was functional disability as measured by the Shoulder Pain and Disability Index (SPADI). In addition, the secondary outcomes included pain intensity measured using the Numeric Pain Rating Scale (NPRS), active shoulder range of motion (ROM), and the Patient Global Impression of Change (PGIC).

#### Primary outcome measures

Shoulder pain and disability index (SPADI)- The internal consistency of the SPADI was satisfactory for pain (α = 0.859) and disability (α = 0.895). Moreover, a high test–retest reliability value was found for ICC for pain = 0.989 [95% CI = 0.975–0.995]; ICC for disability = 0.990 [95% CI = 0.988–0.998] [18].

#### Secondary outcome measures

Numeric pain rating scale(NPRS)—The test–retest reliability of the NPRS for literate and illiterate rheumatoid arthritis patients is 0.96 and 0.95, respectively. Construct validity of the NPRS by correlation with VAS has a range of 0.86 to 0.95, showing that this measure possesses a high degree of reliability and validity for the measurement of pain intensity [19].

##### Joint range of motion

D WALL to measure shoulder joint ROM. The D-wall platform uses a 3D camera to monitor the joint angles and range of motion. In addition, it provides a reliable measurement and accurate angles. The D-Wall Techno Body is equipped with sensors that are placed at specific points on the body. These sensors tracked the movements of the joints being evaluated. As the user moves the joint through its full range of motion, the sensors capture the data and transmit it to the device software [20].

Evidence supports the validity and reliability of the TecnoBody D-Wall and Kinovea systems for assessing joint ROM during functional movements, with ICC values exceeding 0.90, indicating excellent agreement. The D-Wall system’s 3D motion capture capabilities and real-time biofeedback allow clinicians to track movement patterns accurately, even in complex or multi-joint motions, which can help mitigate errors caused by compensatory movements [21]. To enhance measurement reliability in patients with capsular restriction patterns characteristic of frozen shoulder, a standardized calibration protocol will be performed prior to each assessment session. Participants are instructed to minimize compensatory scapular elevation, and assessor-guided stabilization will be applied when required.

##### Patient Global Impression of Change Scale(PGICS)

The PGICS is a single-item rating scale that has emerged as a reliable and interpretable single-item rating of global improvement in clinical trials for chronic pain. Respondents rate their overall change in condition on a 7-point Likert scale ranging from "very much improved" to "very much worse." Owing to its ease of administration and capacity to capture patient-reported outcomes, the PGICS has provided valuable insights into recent research involving chronic pain [22].

### Anticipated dates of trial commencement and completion

The study started in January 2025 and is scheduled to be completed by July 2026.

### Sample size calculation

As the study is a pilot study, the sample size will depend on the number of patients outflow to the Thumbay Hospital and rehabilitation hospital, Ajman. As for estimated we have decided 12 patients in each group as a thumb rule of pilot study [23].

### Data collection and management

Outcome data will be systematically collected by the primary investigator before and after intervention. These data will be carefully analysed to assess the effects of the intervention. The study will be closely supervised by a professor of physiotherapy who will oversee the data collection process conducted by the primary investigator. To promote participant compliance, timely text message reminders with relevant information about upcoming appointment sessions will be sent to their mobile phones. Regarding data management, all research-related data will be stored securely in a protected database to ensure confidentiality and integrity. Hard copies of the signed informed consent forms will be maintained within the study facility. Monthly backups of all digital data entries will be created and maintained throughout the trial. At the conclusion of the study, the final Excel spreadsheet will be submitted to a statistician for data analysis.

### Statistical analysis

Statistical analysis will be done using an SPSS software where Percentage, Mean, median and mode will be used for descriptive analysis and for comparing continuous outcomes like ROM, NPRS and SPADI, if the data is normally distributed, a One-Way ANOVA be used to assess differences across the three groups, followed by post-hoc for pairwise comparisons. For non-normal data, the Kruskal–Wallis test is appropriate, followed by Mann–Whitney tests to identify significant pairwise differences. A Kruskal–Wallis H test was conducted to compare the mean ranks of PGIC scores among the SSMP, MWM, and sham groups. TENS exposure will be included as a covariate in the primary outcome analyses.

A sensitivity analysis will be conducted to evaluate the potential influence of TENS on pain (NPRS) and functional (SPADI) outcomes across groups. As this study is designed as a pilot randomized controlled trial, the analysis will primarily focus on estimating preliminary effect sizes and confidence intervals to inform the design and sample size calculation of a future definitive trial.

### Dissemination

This work will be presented in International Conference Proceedings and published in an indexed journal.

### Study status

Recruiting.

## Discussion

The management of frozen shoulder remains clinically challenging due to its multifactorial etiology and variable course. Movement-based therapies, particularly Mobilization with Movement (MWM) and the Shoulder Symptom Modification Procedure (SSMP), have emerged as promising alternatives. MWM, developed by Mulligan, has shown superior efficacy in improving range of motion (ROM), pain, and function across numerous randomized controlled trials and systematic reviews for patients with adhesive capsulitis [11, 24–26]. Further, a meta-analysis reinforces MWM as a reliable and well-tolerated intervention for this clinical condition [27] with the evidence to produce desirable effect size on outcomes [28]. On the other hand, SSMP, a newer approach by Lewis, emphasizes symptom modification through posture and movement corrections for shoulder conditions. While it offers individualized, non-invasive symptom relief, its clinical reliability and mechanisms remain under-researched for adhesive capsulitis of shoulder.

Both SSMP and MWM operate within a neurophysiological and biomechanical framework that emphasizes pain modulation and movement retraining [29]. MWM appears to reduce discomfort and restore range of motion by activating joint mechanoreceptors and central pain inhibitory pathways, while simultaneously facilitating collagen fibres realignment and promoting ligamentous extensibility along the lines of stress [30]. In contrast, SSMP provides an individualized, patient-centred approach by identifying and addressing specific movement and postural contributors to pain, allowing tailored interventions that respect patient tolerance and functional needs. Meanwhile, sham mobilization serves as an important control in clinical trials, highlighting the role of patient expectations and therapeutic context in manual therapy outcomes.

As the first study to investigate SSMP in frozen shoulder population, it aims to assess the feasibility, acceptability, and procedural fidelity of the symptom modification interventions, along with recruitment strategies using AC scale and patient adherence. It will provide preliminary effect size estimates for pain (NPRS), disability (SPADI), range of motion (ROM), and patient-reported outcomes (PGICS), informing sample size calculation for a future large-scale trial. The inclusion of sham mobilization will clarify whether observed improvements are due to specific therapeutic effects or nonspecific contextual factors critical for interpreting manual therapy outcomes in frozen shoulder rehabilitation.

## Supplementary Information

Supplementary Material 1.

## Data Availability

No data are associated with this article.
