# Supplementary material for: Effectiveness of shoulder symptom modification approaches in managing patients with frozen shoulder: study protocol for a randomized sham-controlled trial
Source: Trials. 2026 Apr 16;27:395. doi: 10.1186/s13063-026-09661-z (PMC13202908; doi:10.1186/s13063-026-09661-z)
Supplement: Supplementary file 1 — Supplementary Material 1. [file 13063_2026_9661_MOESM1_ESM.docx]

Annexure 1. SPIRIT checklist


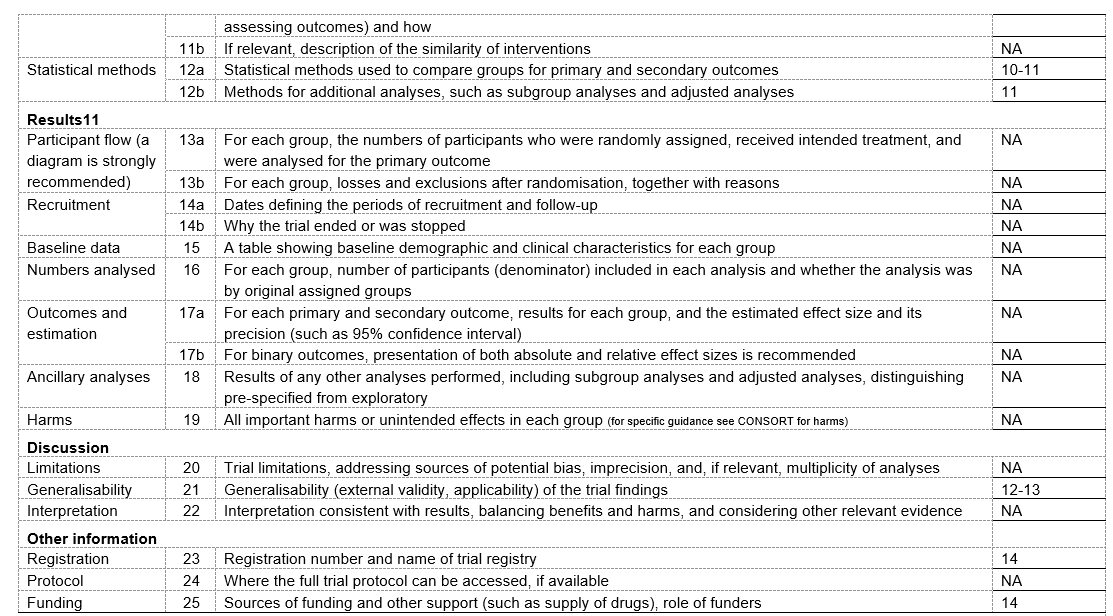

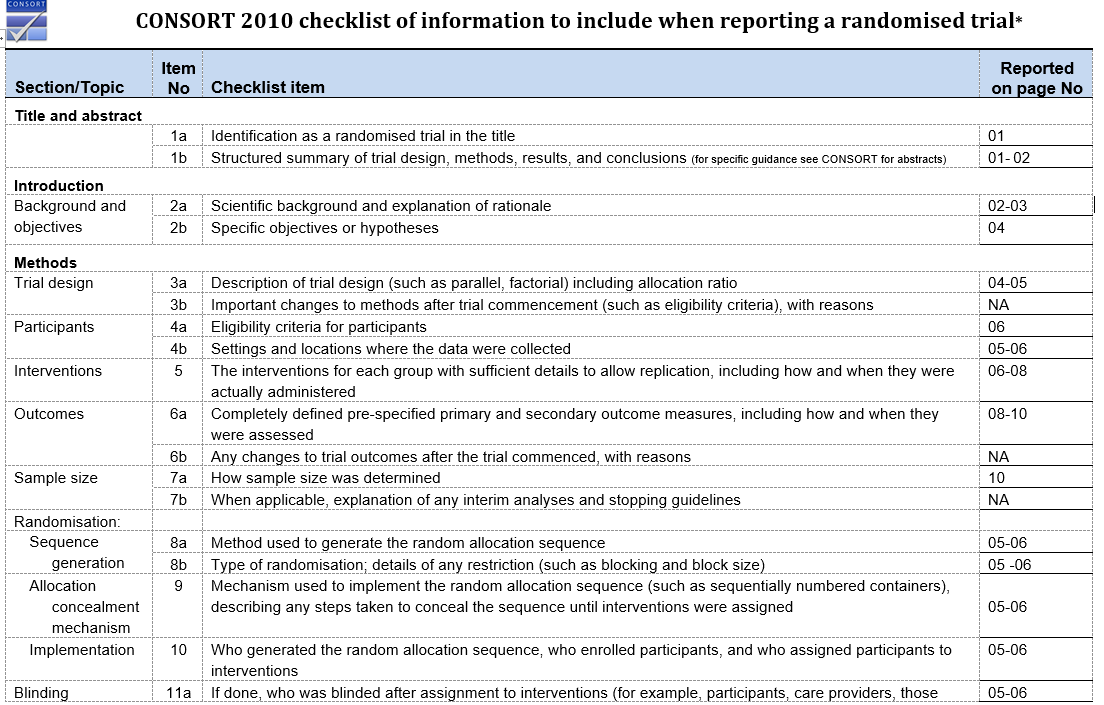


|  | **Enrolment** | **Allocation** | **Post- allocation** | | | | **Close-out** | | | |
| --- | --- | --- | --- | --- | --- | --- | --- | --- | --- | --- |
| **TIMEPOINT**** | ***-t_1_*** | **0** | ***Week 4*** | | ***Week 6*** | ***Week 8*** | | ***t_x_*** | |  |
| **ENROLMENT:** |  |  |  | |  |  | |  | |  |
| **Eligibility screen** | X |  |  | |  |  | |  | |  |
| **Informed consent** | X |  |  | |  |  | |  | |  |
| **Baseline Assessment** | X |  |  | |  |  | |  | |  |
| **Allocation** |  | X |  | |  |  | |  | |  |
| **INTERVENTIONS:** |  |  |  | |  |  | |  | |  |
| **SSMP** |  |  |  |  |  |  |  |  | |  |
|  |  |  |  |  |  |  |  |  |  |  |
| **MWM** |  |  |  | |  |  | |  | |  |
| **Sham Mobilization** |  |  |  |  |  |  | |  |  |  |
|  |  |  |  |  |  |  | |  |  |  |
| **ASSESSMENTS:** |  |  |  | |  |  | |  | |  |
| ***NPRS*** | X |  | X | | X | X | | X | |  |
| ***SPADI%*** |  | X | X | | X | X | | X | |  |
| ***ROM of Shoulder*** | X |  | X | | X | X | | X | |  |
| ***PGIC*** |  |  |  | |  |  | | X | |  |

Annexure 2
